# The Sinai Robotic Surgery Trial in HPV-related oropharyngeal squamous cell carcinoma (SIRS 2.0 trial) – study protocol for a phase II non-randomized non-inferiority trial

**DOI:** 10.3389/fonc.2022.965578

**Published:** 2022-08-25

**Authors:** Raymond L. Chai, Rocco M. Ferrandino, Christine Barron, Kianoush Donboli, Scott A. Roof, Mohemmed N. Khan, Marita S. Teng, Marshall R. Posner, Richard L. Bakst, Eric M. Genden

**Affiliations:** ^1^ Department of Otolaryngology – Head and Neck Surgery, Icahn School of Medicine at Mount Sinai, New York, NY, United States; ^2^ Department of Hematology/Oncology, Icahn School of Medicine at Mount Sinai, New York, NY, United States; ^3^ Department of Radiation Oncology, Icahn School of Medicine at Mount Sinai, New York, NY, United States

**Keywords:** de-escalation, human papillomavirus, oropharynx cancer, transoral robotic surgery, circulating tumor DNA (ctDNA)

## Abstract

**Background:**

Human papillomavirus associated oropharyngeal squamous cell carcinoma (HPVOPSCC) usually affects a younger patient population. As such, the risk for long term toxicity associated with therapy is an important consideration. Multiple trials focused on de-escalation of therapy to preserve survival outcomes while minimizing treatment toxicity are currently in progress, however the question of which patients are ideal candidates for de-escalation remains unanswered. Circulating tumor DNA (cfHPVDNA) has emerged as a means of monitoring disease in patients with HPVOPSCC. Undetectable postoperative cfHPVDNA levels portend a better prognosis and by extension, may identify ideal candidates for de-escalation therapy. We propose an overview and rationale for a new institutional clinical trial protocol focusing on the use of cfHPVDNA to risk stratify patients for adjuvant therapy. We hypothesize that many surgical patients currently receiving radiation therapy may be clinically observed without adjuvant therapy.

**Methods:**

Patients with measurable cfHPVDNA and clinically resectable HPVOPSCC will undergo TORS resection of tumors and neck dissection. Patients with undetectable cfHPVDNA at 3 weeks post-op will be allocated to low or high-risk treatment protocol groups. The low risk group consists of patients with <4 positive lymph nodes, ≤2 mm extranodal extension (ENE), and perineural invasion (PNI) or lymphovascular invasion (LVI) alone. The high-risk group is made up of patients with ≥4 positive lymph nodes, gross ENE, positive margins, N2c disease and/or the combination of both PNI and LVI. The low-risk group will be allocated to an observation arm, while the high-risk group will receive 46 Gy of adjuvant radiotherapy and weekly cisplatin therapy. The primary outcome of interest is 2-year disease recurrence with secondary outcomes of 2-year disease free survival, locoregional control, overall survival, and quality of life measures. A sample of 126 patients in the low-risk group and 73 patients in the high-risk group will be required to evaluate non-inferiority to the standard of care.

**Discussion:**

This study will provide much needed recurrence and survival data for patients that undergo primary TORS followed by observation or de-escalated adjuvant therapy. Additionally, it will help delineate the role of cfHPVDNA in the risk stratification of patients that undergo treatment de-intensification.

## Background

### Prognosis of HPV–related oropharynx cancer

HPV-related oropharyngeal squamous cell carcinoma (HPVOPSCC) now accounts for over 80% of oropharyngeal carcinoma seen in the United States and an increasing fraction of these malignancies in Europe (1–3). The demographics and excellent prognosis of HPVOPSCC has called into question the rationale for aggressive concurrent chemoradiotherapy (CRT) for early or intermediate staged disease. Studies in unselected patients suggest that patients with HPVOPSCC have a better prognosis than patients with HPV-negative, predominantly environmentally related oropharyngeal squamous cell carcinoma (EROPC) (4–9). Subgroup analyses of several Phase III randomized clinical trials in locally advanced head and neck cancer (HNC) continue to demonstrate the prognostic importance of HPV status. In these trials, HPVOPSCC patients consistently demonstrated improved overall survival, progression free survival, and locoregional control compared to patients with EROPC (6–9).

### Functional and oncologic outcomes of transoral surgery versus CRT for HPV-related oropharynx cancer

Given the excellent prognosis of HPV-related oropharyngeal cancers, emphasis has shifted to reducing the morbidity associated with therapy while maintaining survival. Many of the functional limitations incurred by CRT are related to long term toxicities. One retrospective analysis of three RTOG trials suggested that the rate of severe late toxicities in patients receiving CRT is 35% for patients with oropharyngeal cancer (10). Another prospective study of 104 patients (72 oropharyngeal cancers) found that 26.4% of patients are feeding tube dependent at one year, and 13.8% were tracheotomy dependent (although less commonly for oropharyngeal cancers) (11).

Proponents of transoral robotic surgery (TORS) for oropharynx cancer contend that it offers improved functional outcomes when compared to non-surgical treatment with radiation therapy with or without chemotherapy. Multiple studies report favorable swallowing outcomes using TORS for resection of oropharyngeal cancers (12–18). Additionally, the phase 2 randomized ORATOR and ORATOR2 trials demonstrated similar MDADI scores for the RT and TORS treatment arms of their studies up to 3 years of follow up (19, 20). Taken together, patient reported quality-of-life is at least comparable, if not better, for TORS when compared to CRT.

Finally, the retrospective oncologic outcomes from TORS surgery for oropharyngeal cancer have demonstrated favorable results. Several studies have reported local failure rates which varied between 0-3% with median follow-up rates ranging from 18 months to two years (12, 18, 21, 22). Regional recurrence rates in the same studies varied between 2-8%, while distant disease was identified in 1-9% of patients (12, 18, 21, 22). Eighteen month overall survival was 90% in one study (18), and two-year overall survival was 82% and 80.6% in two other studies (12, 21). Furthermore, in patients undergoing TORS and selective neck dissection, pathologic data can provide staging information that may allow for tailoring treatment to the individual and may potentially obviate the need for unnecessary toxic adjuvant treatments. Additionally, patients with unfavorable pathologic criteria are identified and more intense adjuvant therapy may be applied more appropriately.

These findings compared favorably to existing reports of oropharyngeal cancer treated with CRT (23–27). There is definitive evidence that radiation effects on critical structures such as the pharyngeal constrictors are dose dependent, and reduced dosage will reduce the risk of chronic dysphagia, feeding tube dependence, and aspiration risk (28–31). By physically removing tumor, one can theoretically offer a lower dose or entirely eliminate primary radiation reserving it for salvage and confer better swallowing and quality of life (QOL) outcomes.

### De-escalation of therapy for early-stage HPV-related OPSCC

In general, patients with HPVOPSCC are young and will live for prolonged periods. They are at high-risk for long-term toxicity and mortality from therapy (10). While the long-term consequences of chemotherapy for head and neck cancer are relatively constrained, high-dose radiation therapy (RT) and CRT substantially impact on local tissues and organ function and result in a significant rate of late mortality and morbidity in patients (32–35). Studies are now being designed to reduce the impact of RT and CRT for patients.

There are currently few trials examining the role of de-escalation using surgery alone in early T-stage HPV-related disease or reduced RT in intermediate risk patients. New surgical techniques have broadened the range of patients capable of achieving a complete resection and the functional outcomes in such patients. Furthermore, the sensitivity of HPVOPSCC to chemotherapy and RT raise the possibility that delayed or salvage treatment in early-stage patients would be highly effective, would result in similar survival outcomes and could be applied to a much smaller population than current standards call for. Looked at from a different perspective, the need for post-operative RT in this younger, HPV+ and more functional population have not been validated in clinical trials to date.

The recently published non-randomized phase II Sinai Robotic Surgery (SIRS) trial by our group (NCT02072148) demonstrated the highly favorable oncologic outcomes with de-intensified risk stratified postoperative adjuvant treatment following TORS (36). Here, with a median follow-up time of 43.9 months, DSS was 100% in the study cohort while PFS was 90.7%. There was no statistical difference in oncologic outcomes between the surgery alone, adjuvant radiation, and adjuvant chemoradiation groups. Of the four failures presenting with locoregional recurrence alone without distant disease, all were successfully salvaged and remained disease-free with a minimum follow-up time of 11 months. In the low-risk group receiving no adjuvant therapy following surgery, PFS was 91.3%. In addition, ECOG 3311 recently published three year follow up results demonstrating PFS of 96.9% for the 38 low-risk patients treated with TORS alone (37).

### cfHPVDNA stratification

Cell-free HPV DNA (cfHPVDNA) has emerged as a promising biomarker for HPVOPSCC (38). Dynamic changes in cfHPVDNA correlate with both treatment response in patients with either localized or metastatic HPVOPSCC as well as surveillance for cancer recurrence after curative intent therapy (38–41). Prior data suggests that a negative test in the surveillance period following definitive treatment is highly sensitive and specific for recurrent disease (100% NPV) (38). A potential benefit of cfHPVDNA may be in treatment personalization by stratifying patients into risk groups to determine appropriate treatment intensity. Patients with low-risk disease, identified by undetectable cfHPVDNA following surgery, may be amenable to reduction or elimination of adjuvant therapy.

There is also emerging evidence that this prognostic information can be used to risk stratify patients for personalized therapy in other head and neck cancers. In a modern cohort treated with intensity modulated radiation therapy, patients with nasopharyngeal carcinoma with high Epstein-Barr virus (EBV) cfEBV DNA levels displayed superior overall survival and distant metastasis-free survival with the addition of concurrent chemotherapy while patients with lower EBV cfEBV DNA levels did not derive apparent benefits from chemotherapy (42). Similarly, for patients receiving induction chemotherapy followed by chemoradiation, detectable cfEBV DNA throughout multiple time points was strongly associated with inferior outcomes and enabled the definition of distinct risk groups based on baseline cfEBV DNA post therapy kinetics (43).

Multiple trials are underway to evaluate the role of de-intensification of therapy, including varied approach such as decreasing radiation dose, limiting chemotherapy dose, and upfront surgery followed by risk-stratified adjuvant therapy (44). Amidst these trials, there is a clear need to study the use of surgery alone to replace radiation for LRC, reserving salvage CRT for locoregional failure and sparing a large fraction of patients the morbidity and mortality of RT. In this study we plan to investigate the impact of de-escalated therapy on locoregional recurrence rates. Additionally, we hope to elucidate the utility of cfHPVDNA in risk stratification for therapy and surveillance.

## Methods/Design

**Figure 1:**
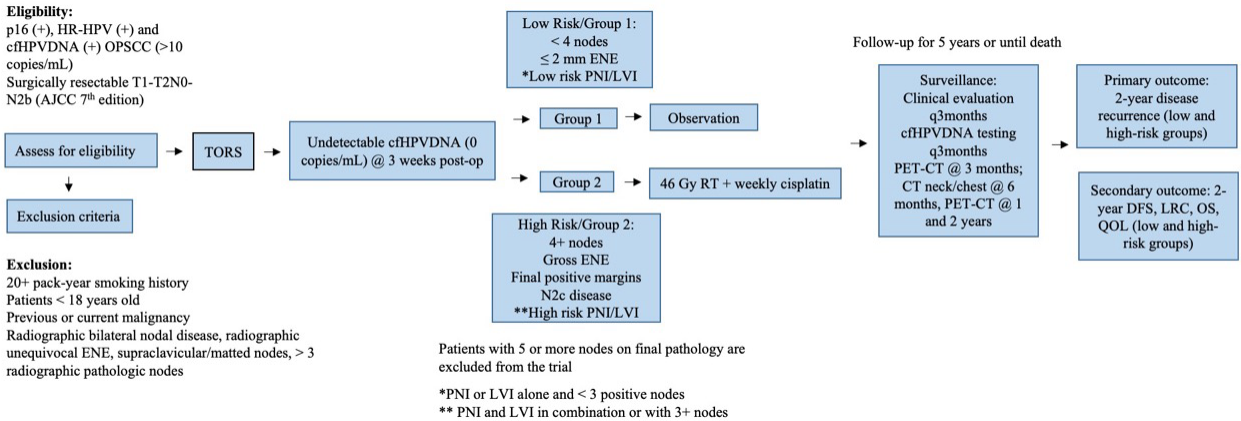
Protocol Schematic.

The SIRS 2.0 trial (NCT05419089) is a non-randomized Phase II non-inferiority trial approved by the Institutional Review Board of the Icahn School of Medicine at Mount Sinai (**Figure 1**).

### Objectives

To test the non-inferiority of 2-year recurrence rates in patients with HR-HPV and p16+-related OPSCC treated with surgery alone or surgery with de-intensified adjuvant therapy when compared to current regimens involving standardized post-operative radiotherapy +/- chemotherapy. The secondary objectives include determination of 2-year DFS, LRC, OS, and quality of life outcomes for patients treated with surgery alone and surgery with de-intensified adjuvant therapy.

### Primary endpoint

● The proportion of early stage HPVOPSCC patients treated with surgery alone having undetectable cfHPVDNA after surgery showing LRR at 2 years (Group 1)
● The proportion of HPVOPSCC patients treated with surgery and de-intensified adjuvant protocol having undetectable cfHPVDNA after surgery showing LRR at 2 years (Group 2)

### Secondary endpoints

● PFS at 2 years is defined as the proportion of patients without events (recurrence or death) at 2 years after surgery (Group 1, low-risk group)
● DFS at 2 years is defined as the proportion of patients without events (any recurrence – including local, regional, or distant recurrence) at 2 years after surgery (Group 1, low-risk group).
● OS at 2 years is defined as the proportion of patients alive at 2 years after surgery (Group 1, low-risk group)
● The score of QOL questionnaires (Group 1, low-risk group).
● PFS at 2 years is defined as the proportion of patients without events (recurrence or death) at 2 years after adjuvant CRT (Group 2, high-risk group)
● DFS at 2 years is defined as the proportion of patients without events (any recurrence – including local, regional, or distant recurrence) at 2 years after adjuvant CRT (Group 2, high-risk group).
● OS at 2 years is defined as the proportion of patients alive at 2 years after adjuvant CRT (Group 2, high-risk group)
● The score of QOL questionnaires after adjuvant CRT (Group 2, high-risk group)
● The frequency, percentage, and measured values of biomarkers present in patients with locoregional and distant failure.

### Inclusion criteria

● Histologically or cytologically confirmed and identified resectable primary OPSCC with p16+ IHC
● Pre-surgery detectable (≥10 copies/ml) baseline cfHPVDNA using NavDX assay (Naveris, Cambridge, MA). Undetectable cfHPVDNA at 3 weeks postoperatively.
● AJCC 7^th^ edition early and intermediate stage disease (T1N0-2B, T2N0-2B) (non-matted) disease without evidence of distant metastases or gross extranodal extension.
● Age ≥ 18 years
● No previous surgery, radiation therapy, or chemotherapy for head and neck cancer (other than excision/incisional biopsy of the primary site, excisional/incisional nodal biopsy, or tonsillectomy) is allowed at time of study entry.
● Eastern Cooperative Oncology Group (ECOG) performance status of 0 or 1.
● No active tobacco use (≥1 cigarette or cigarette-equivalent per day within the last 5 years) and no cumulative smoking history of > 20 pack years.
● Ability to understand and willingness to sign a written informed consent document.
● Good Bone marrow, hepatic and renal function, defined as: Neutrophil count ≥ 1.5 x 10^9^/l, Platelet count ≥ 100 x 10^9^/l, Hemoglobin ≥ 10 g/dl (may achieve by transfusion), Renal function: ≥ 60 ml/min (actual or calculated by the Cockcroft-Gault method, or a creatinine ≤ the upper limits of normal.

### Exclusion criteria

● Age < 18 years.
● Pregnant or breast-feeding women.
● Previous or current malignancies at other sites, except for adequately treated *in situ* carcinoma of the cervix, basal or squamous cell carcinoma of the skin, thyroid cancer, prostate cancer treated with surgery/radiotherapy, or other cancer curatively treated by surgery and with no current evidence of disease for at least 5 years.
● Other serious illnesses or medical conditions that may be contraindications to radiotherapy, chemotherapy, or surgery.
● Advanced nodal stage (AJCC 7^th^ edition N2C, N3) or surgically unresectable disease or disease that cannot be fully resected, unequivocal radiographic extranodal extension, supraclavicular or matted metastatic disease, >3 radiographic pathologic cervical nodes.
● Non-HR-HPV subtype on initial biopsy or final pathology.
● 5 or more positive nodes, irrespective of size, on final pathology.
● p16 or HPV negative OPSCC as determined by IHC and PCR, respectively.
● Undetectable or < 10 copies/mL baseline cfHPVDNA prior to surgery
● Autoimmune disease treated with chemotherapy agents, anti TNF agents, or hydroxychloroquine within the last 5 years

### Patient evaluation

The following evaluations are required prior to screening:

● History and physical examination including medical history, smoking history, current medications, and flexible laryngoscopy will be performed in a multidisciplinary head and neck cancer clinic.

The following evaluations are required prior to surgery:

● Obtain written informed consent to participate in study
● Complete screening and eligibility checklist
● Preoperative cfHPVDNA testing
● Complete blood count and basic metabolic panel testing
● Dysphagia assessment questionnaires
● B-HC (for WOCP) within 7 days of registration
● PET/CT or high-resolution CT or MRI of the neck within 28 days before registration.
● Clinical TNM staging
● Examination under anesthesia within 8 weeks or at time of surgery

The following evaluations are required prior to adjuvant treatment group assignment:

● Pathologic evaluation of surgical specimens including p16 IHC and HR-HPV PCR, margin status in mm, PNI, LVI, ENE defined as ≤ or > 2mm, number of cervical metastatic nodes, location of involved nodes, and pAJCC staging.
● 3-week postoperative cfHPVDNA testing

### Treatment

#### Surgery

Eligible patients will undergo TORS resection of the tumor with negative intraoperative frozen section margins. Ipsilateral levels II-IV selective neck dissection will be routinely performed. Contralateral neck dissection, additional neck dissection levels (i.e. I,V), and appropriate reconstruction will be performed at the discretion of the operating surgeon. Surgery is to proceed until negative final frozen section margins are obtained.

### Low-risk

Patients will be assigned to the low-risk group 1 if they meet the following criteria:

● Complete resection to negative frozen section margins (pT1-2)
● ≤ 3 nodes, ≤ 2 mm extranodal extension (ENE), no supraclavicular nodes
● HR-HPV positive
● Undetectable cfHPVDNA at 3 weeks post-operatively

#### Group 1 - observation protocol

Patients assigned to the low-risk group 1 will treated and monitored as outlined below:

● No adjuvant therapy
● Clinical evaluations every 3 months the first year
● PET-CT at 3 months post-surgery.
● CT neck and chest at 6 months.
● PET-CT at 1 year and 2 years post-operatively unless clinically indicated.
● Imaging to be supplemented with HPV cfHPVDNA testing 12 weeks after surgery and every 3 months for the first two years after surgery.
● If cfHPVDNA test is positive, patient will have repeat testing 4 weeks later. If test continues to be positive, this finding will mandate an enhanced PET-CT. For patients with two consecutive positive cfHPVDNA tests post-operatively and no obvious primary identified on clinical examination and imaging, both enhanced PET-CT and clinical examination will be performed every 3 months until the emergence of a defined site of recurrence. If no obvious recurrence after 6 months of routine testing and examination, timing of further clinical evaluation and imaging will be relegated to investigator’s choice.

### High-risk

Patients will be assigned to the high-risk group 2 if they meet the following criteria:

● The presence of any of the following: 4 positive nodes, gross ENE, final positive margins, or bilateral neck disease
● High-risk PNI/LVI (defined as PNI and LVI in combination or either factor in the presence of 3 or more positive nodes)
● Undetectable cfHPVDNA at 3 weeks post-operatively

#### Group 2 – de-intensified adjuvant protocol

Patients assigned to the high-risk group 2 will treated and monitored as outlined below:

● Postoperative chemoradiation (4600 cGy with weekly cisplatin for a total dose of 200 mg/M^2^)
● Clinical evaluations every 3 months during the first year
● PET-CT at 3 months post-surgery.
● CT neck and chest at 6 months.
● PET-CT at 1 year and 2 years post-operatively unless clinically indicated.
● Imaging to be supplemented with cfHPVDNA testing at 6 and 12 weeks after CRT is completed and every 3 months for the first two years after CRT. If positive, will get repeat testing 4 weeks later. If continues to be positive, will mandate enhanced PET-CT. If no obvious recurrence after 6 months of routine testing and examination, timing of further clinical evaluation and imaging will be relegated to investigator’s choice.
● Patients with detectable/positive cfHPVDNA post-operatively will be excluded from the study. They will be offered a separate sub-study
● Patients with 5 or more pathologic nodes on final pathology will be excluded from the study, irrespective of cfHPVDNA status and will be offered a separate sub-study.

### Radiation

All patients will receive daily radiation treatment with either intensity-modulated radiotherapy (IMRT) or proton beam therapy. Photon beams of ≥4 MV are required. Patients not receiving proton therapy are required to receive Intensity Modulated Radiotherapy (IMRT) is required for all cases. IMRT *via* dynamically moving leaves, step-and-shoot with a multileaf collimator, Rapid Arc, binary multileaf collimator and tomotherapy are allowed. Proton beam therapy will be delivered with either passive scattered, uniform scanning beam, pencil beam scanning (PBS), which is also called intensity modulated proton therapy (IMPT) techniques depending on the facility’s experience and equipment.

### Immobilization, simulation, and localization

A thermoplastic head mask (or similar immobilization device) is required for IMRT or proton beam. A treatment planning CT scan is mandatory for defining target volumes, however IV contrast is not required. Volumes will be contoured on each relevant CT slice (≤0.3 cm thickness).

### Radiotherapy volume definitions

#### Group II

Since radiotherapy will only be administered postoperatively, there should not be gross tumor volume (GTV). Clinical target volume (CTV) is generated to account for microscopic disease. CTV46 will include the primary tumor bed and/or at-risk lymph node regions in the neck (excluded bone and air). The planning target volume (PTV) includes a 0.5 cm expansion margin that accounts for daily setup variation. For proton planning, the worst case CTV dose distribution corresponding to a setup error of 3 mm and 3% range uncertainty will be used for dose optimization, evaluation and dose reporting in place of PTV.

### Dose constraints

Target dose constraints and avoidance structures are shown in **Table 1**.

**Table 1:** Radiation dose constraints for avoidance structures.

Structure	Dose Constraints
Spinal Cord	Maximum dose <40 Gy
Expanded spinal cord (5mm expansion around spinal cord)	Maximum dose <45 Gy
Brainstem	Maximum dose <50 Gy
Parotid Glands	Mean dose <26 Gy
Larynx	Mean dose <40 Gy
Mandible	Maximum dose <60 Gy
Oral Cavity	Mean Dose <40 Gy
Inner Ear	Mean Dose <50 Gy
Optic nerves	Maximum Dose <46 Gy
Optic Chiasm	Maximum Dose <46 Gy
Expanded Optic Nerve and chiasm (2 mm expansion around structures)	Maximum Dose <54 Gy
Retina	Maximum dose <45 Gy
Brain	Maximum dose <54 Gy

### Radiotherapy planning

All plans must be normalized such that 95% of the PTV is covered with the prescription dose. No more than 20% of the PTV may receive > 110% of the prescription dose. No more than 1% of the PTV may receive < 93% of the prescription dose. No more than 1cc of tissue outside the PTV may receive > 110% of the prescription dose.

For proton planning, each beam has an individual and unique expansion from the CTV especially along the beam direction. When the robust optimization is employed, the optimization target volume is determined from CTV based on beam arrangement and will take into account proton range uncertainty as well. For dose reporting, CTV+5mm can be utilized as the PTV; however, for proton plans the plan should always be optimized to the CTV.

### Radiotherapy quality assurance

The following quality assurance measures will be implemented to ensure effective treatment and patient safety.

● On the first day of treatment each patient will have electronic portal images taken that localize the isocenter placement. For proton plans, quality assurance is performed by delivering the plan onto a phantom and measuring the dose using an ion chamber array or similar device.
● Every week, verification portal images are taken and approved by a radiation oncologist prior to continuation of treatment. For PBRT patients, weekly verification CT or conebeam CT scans are required and any plan adaptations will be determined accordingly.
● Each treatment plan will be reviewed by an non-treating radiation oncologist prior to therapy to assure appropriate fields and doses.

### Concurrent chemotherapy

Patients in Group 2 will also receive weekly cisplatin at a dose of 40mg/M^2^/week for a total dose of 200 mg/M^2^. Patients will be prehydrated with 1 liter normal saline before and after. The prehydration liter will include a 5HT3 antagonist as part of the antiemetic regimen. 20 Meq KCl and 2 gm Magnesium sulfate will be administered in the post hydration liter. In the event of toxicity to cisplatin, carboplatin may be substituted as weekly chemotherapy with AUC of 2.0.

### Follow up evaluation and assessment of efficacy

Participants will be followed for 5 years after completion of the study treatment or until death.

Group 1 and Group 2 patients will have the following recurrence monitoring protocol:

● Clinical evaluations every 3 months the first year
● PET-CT at 3 months post-surgery.
● CT neck and chest at 6 months.
● PET-CT at 1 year and 2 years post-operatively unless clinically indicated.
● Imaging to be supplemented with cfHPVDNA testing 12 weeks after surgery for Group 1 and 6 and 12 weeks after surgery for Group 2. Participants will also get cfHPVDNA testing every 3 months for the first two years after surgery.
● If cfHPVDNA test is positive, patient will have repeat testing 4 weeks later. If test continues to be positive, this finding will mandate an enhanced PET-CT. For patients with two consecutive positive cfHPVDNA tests post-operatively and no obvious primary identified on clinical examination and imaging, both enhanced PET-CT and clinical examination will be performed every 3 months until the emergence of a defined site of recurrence. If no obvious recurrence after 6 months of routine testing and examination, timing of further clinical evaluation and imaging will be relegated to investigator’s choice.

### Measurement of Effect

Survival and recurrence data will be tracked for five years from surgery. Quality of life measures including the European Organization for Research and Treatment of Cancer Core measure (EORTC QLQ-C30) and the head and neck-specific measurement tool EORTC QLQ H&N35, M.D. Anderson Symptom Inventory – Head and Neck (MDASI-HN), and MDADI-HN dysphagia and xerostomia questions will be assessed at the 3-, 6-, and 12-month time points and then yearly for 5 years. Toxicity evaluation for both arms will be assessed starting from the time of surgery.

### Statistics

#### Sample size calculation and accrual targets

Sample sizes for the two study arms were calculated separately. For the low-risk group, assuming a historical recurrence proportion of 15%, a non-inferiority margin of 11%, one interim analysis, and 10% dropout rate, a total sample of 126 patients will be enrolled to provide power of 83.3% and one-sided alpha of 0.025. For the high-risk group, assuming the same recurrence proportion, a non-inferiority margin of 15%, one interim analysis, and 30% dropout rate, a sample of 73 patients will be enrolled to provide 82.5% power and one-sided alpha of 0.05%. An interim analysis will be performed after 57 and 26 patients are recruited and followed for 2 years for the low-risk and high-risk groups, respectively. We expect to accrue 5 patients per month for both treatment arms with 80% allocating to the low-risk group.

#### Analysis plan

Participant characteristics will be summarized using descriptive statistics. The analysis for the primary and secondary endpoints will be performed for the low- and high-risk groups separately using intention-to-treat (ITT) and per-protocol cohorts, respectively (low-risk group: ITT; high-risk group: ITT+PP). The primary endpoint of proportion of recurrence for the low- and high-risk groups will be reported with corresponding 97.5% and 95% upper confidence limits. The Kaplan-Meier (KM) method will be used to estimate locoregional control (LRC), disease-free survival (DFS), and overall survival (OS). Patients without the designated event will be censored at the time of the last available follow-up record or the study analysis cutoff time point. Quality of life outcomes will be compiled and recorded for each of the outlined time points and linear mixed-effect models will be used to test changes of the scores over time. Biomarker outcomes will be reported descriptively and will be summarized by patient outcomes.

## Discussion

HPV-related oropharyngeal cancers have demonstrated a significantly better prognosis compared to non-HPV-related cancers. Given the excellent prognosis, focus has shifted to reducing the morbidity associated with therapy while maintaining survival. In the original Sinai Robotic Surgery trial, patients treated with surgery alone maintained a >90% progression free survival at a median of 43 month follow up. In this study we plan to evaluate the non-inferiority of 2-year locoregional recurrence rates as well as survival outcomes for low-risk patients treated with TORS alone and high-risk patients treated with TORS and reduced dose adjuvant chemoradiotherapy with undetectable postoperative cfHPVDNA. The objective of this study is to determine whether or not comparable survival outcomes can be achieved in patients treated with de-escalated adjuvant therapy. Additionally, we plan to evaluate the impact of the proposed de-escalation protocol on quality of life outcomes.

In this non-inferiority trial, we seek to shed light on the changes in cfHPVDNA levels that occur during treatment and specifically with surgical resection. Recent studies demonstrated that surgically treated patients have diminished levels of cell free tumor DNA in their blood within 24 hours of surgical resection (45). Additionally, Routman *et al.* demonstrated that surgery alone can reduce cfHPVDNA to undetectable levels in 59% of patients (39). We believe that the dynamic changes in cfHPVDNA in the pre-adjuvant period may help to identify candidates for de-escalated treatment, however more longitudinal data is needed to determine its utility for risk stratification. Additionally, the ideal application of cfHPVDNA in monitoring for clinical recurrence has not been fully elucidated. In this study we will obtain cfHPVDNA levels at three-month intervals which we believe may help to identify subclinical recurrence.

At this point, there still remains a question of which treatment modality for early stage HPVOPSCC provides the best functional outcomes. With exceptions including the ECOG 33-11, ORATOR, ORATOR2, and SIRS trials, most of the data related to functional outcomes after TORS has been retrospective in nature. There are a number of prospective clinical trials and observational studies currently underway that seek to elucidate the functional impacts of TORS compared to definitive radiation with or without chemotherapy. NCT02984410 and NCT04124198 are two currently recruiting randomized control trials directly comparing TORS to radiation therapy with a primary focus on functional/quality of life outcomes. NCT04638465, NCT03418909, and NCT05341479 are three prospective observational cohort studies evaluating survival and quality of life outcomes for patients treated with TORS compared to radiation (+/- chemotherapy). Each of these studies will add to the current body of literature, particularly with regard to standard radiation dosing, however there is a growing number of de-escalation studies currently in progress.

The survival differences observed in HPVOPSCC when compared to EROPC motivated the pursuit of de-escalated therapy for these typically more radiosensitive tumors. At present, there are few studies listed on clinicaltrials.gov employing TORS as part of a de-escalation protocol. In addition to our own study, there are two other ongoing single arm prospective studies, NCT04502407 and NCT03729518, recruiting patients to receive reduced dose adjuvant (chemo)radiotherapy after transoral robotic surgical resections with a focus on survival and quality of life outcomes. In contrast, NCT04572100 and NCT03107182 employ neoadjuvant therapy regimens to determine radiologic tumor response before assigning subjects to receive TORS vs (chemo)radiotherapy. Interestingly, NCT04572100 also evaluates the cfHPVDNA changes that occur in response to therapy. Similar to our study, NCT05387915 is enrolling patients with undetectable cfHPVDNA and intermediate pathologic risk factors to receive three weeks of adjuvant radiotherapy after TORS. In contrast to our protocol, the is no observation arm and this study primarily focuses on the evaluation of swallow function after de-intensified therapy, with secondary survival and recurrence outcomes. While this does not represent a comprehensive list of ongoing trials, these protocols highlight the transition occurring in the treatment of HPVOPSCC, with de-escalated therapy using a surgical approach becoming a more common focus of research.

While HPV-related cancers generally confer a favorable prognosis, outliers frequently remain particularly memorable in our minds. In these individuals, the pathologic features and clinical risk factors may not identify these more insidious tumors. As such, discovery of new biomarkers for risk stratification is critical in order to continue tailoring individualized treatment plans. This study represents one of the first prospective clinical trials integrating circulating tumor biomarkers into a de-escalation study for HPVOPSCC. We believe this not only benefits enrolled patients from a morbidity and quality of life standpoint, but also serves to push our field forward toward integration of biomarkers into the treatment decision process.

## Data availability statement

The original contributions presented in the study are included in the article/supplementary material. Further inquiries can be directed to the corresponding author.

## Ethics statement

The studies involving human participants were reviewed and approved by Institutional Review Board of the Mount Sinai School of Medicine. The patients/participants provided their written informed consent to participate in this study.

## Author contributions

All authors listed have made a substantial, direct, and intellectual contribution to the work and approved it for publication.

## Conflict of interest

The authors declare that the research was conducted in the absence of any commercial or financial relationships that could be construed as a potential conflict of interest.

## Publisher’s note

All claims expressed in this article are solely those of the authors and do not necessarily represent those of their affiliated organizations, or those of the publisher, the editors and the reviewers. Any product that may be evaluated in this article, or claim that may be made by its manufacturer, is not guaranteed or endorsed by the publisher.
